# Translational Control of Secretory Proteins in Health and Disease

**DOI:** 10.3390/ijms21072538

**Published:** 2020-04-06

**Authors:** Andrey L. Karamyshev, Elena B. Tikhonova, Zemfira N. Karamysheva

**Affiliations:** 1 Department of Cell Biology and Biochemistry, Texas Tech University Health Sciences Center, Lubbock, TX 79430, USA; elena.tikhonova@ttuhsc.edu; 2 Department of Biological Sciences, Texas Tech University, Lubbock, TX 79409, USA

**Keywords:** protein synthesis, protein transport, signal sequence, signal recognition particle (SRP), protein quality control, translation regulation, RNA degradation, ribosome, human diseases, disease-causing mutations

## Abstract

Secretory proteins are synthesized in a form of precursors with additional sequences at their N-terminal ends called signal peptides. The signal peptides are recognized co-translationally by signal recognition particle (SRP). This interaction leads to targeting to the endoplasmic reticulum (ER) membrane and translocation of the nascent chains into the ER lumen. It was demonstrated recently that in addition to a targeting function, SRP has a novel role in protection of secretory protein mRNAs from degradation. It was also found that the quality of secretory proteins is controlled by the recently discovered Regulation of Aberrant Protein Production (RAPP) pathway. RAPP monitors interactions of polypeptide nascent chains during their synthesis on the ribosomes and specifically degrades their mRNAs if these interactions are abolished due to mutations in the nascent chains or defects in the targeting factor. It was demonstrated that pathological RAPP activation is one of the molecular mechanisms of human diseases associated with defects in the secretory proteins. In this review, we discuss recent progress in understanding of translational control of secretory protein biogenesis on the ribosome and pathological consequences of its dysregulation in human diseases.

## 1. Introduction

Cells synthesize thousands of proteins that have diverse functions and should be directed to specific places in accurate amounts at precise time. In eukaryotes, there are several levels of regulation that direct these processes: RNA synthesis (transcription), splicing, RNA transport, mRNA translation or protein synthesis, protein folding and transport. Although all these processes have high fidelity, mistakes happen and proteins got misfolded and mislocalized because of inherited mutations, or errors during transcription and translation, or loss of the necessary interacting or modifying factors. Many of these aberrant proteins are dangerous for the cell viability because of their toxicity and often are associated with multiple human diseases. Among them are Parkinson’s and Alzheimer diseases, frontotemporal dementia, cystic fibrosis, and many others. Evolution pressure led to evolving protective mechanisms that prevent appearance of defective proteins by destructing the aberrant proteins themselves or sensing the aberrations in the mRNA templates and degrading them. Interestingly, many quality control mechanisms are engaged co-translationally when proteins are being synthesized by the ribosome. It seems that the balance of interactions of the nascent chains on the ribosome during translation is a key element to maintain protein homeostasis in the cells and restrain synthesis of defective proteins [1]. Secretory and membrane proteins represent a special group because many of them have distinct interacting partners on ribosomes that are involved in their targeting and transport. Co-translational targeting of secretory and many membrane proteins to endoplasmic reticulum (ER) occurs via interaction with signal recognition particle (SRP) [2,3], while SRP-independent posttranslational mechanism of targeting involves guided entry of tail-anchored proteins (GET) pathway in yeast and the transmembrane domain recognition complex of 40 kDa (TRC40) pathway in mammals [4,5]. The third SRP-independent pathway recently discovered in yeast is SND (for SRP-independent targeting) pathway, it involves proteins Snd1-3 [6]. It preferentially targets proteins with transmembrane domains located more downstream in contrast to proteins with amino-terminal transmembrane domain being preferential substrates for SRP pathway. SND pathway acts in parallel with SRP and GET and can function as a back-up for both pathways. Thus, SRP, GET and SND pathways act together to ensure efficient targeting of proteins to ER. Systematic analysis of secretome in yeast showed that about 57% of predicted secretory proteins are targeted by SRP and about 43% of secretome are SRP-independent [7]. The SRP-independent pathways and other posttranslational mechanisms of targeting are not in the scope of this review that is focused on SRP-dependent secretory proteins. These secretory proteins are co-translationally recognized by SRP during their synthesis on ribosome and their expression and targeting are regulated during translation. It was demonstrated recently, their quality and expression are controlled during synthesis by a unique mechanism that senses aberrant secretory proteins and degrades their mRNAs preventing synthesis of harmful products [8]. In this review, we analyze and discuss biogenesis of SRP-dependent secretory proteins and role of the protein quality control mechanisms during their synthesis on the ribosome in providing accurate translational control in maintaining secretory protein homeostasis.

## 2. Synthesis and Transport of Secretory Proteins

### 2.1. Secretory Proteins Are Synthesized as Precursors

**Figure 1:**
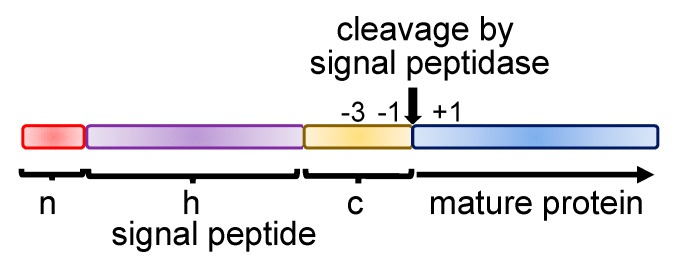
Schematic presentation of a typical signal peptide. Secretory proteins contain an additional sequence at their N-termini named signal peptide or signal sequence. N-terminal portion of the secretory proteins containing a signal peptide and part of a mature protein shown on the scheme. Usually signal peptides include a short positively charged n-region (1–5 amino acid residues) followed by hydrophobic domain (h-region, 7–15 hydrophobic amino acid residues) and c-region with a cleavage site for signal peptidase (3–7 amino acid residues). Positions of the −3, −1, and +1 amino acid residues are shown. See text for details and for the references.

Secretory and membrane proteins represent about 30 to 40 percent of all cellular proteins [9]. Many secretory proteins have a distinctive feature—they are synthesized in a form of precursor with additional sequences at their N-terminal end called signal peptides or signal sequences. Signal peptides serve as labels or tags to mark the proteins that should be transported outside the cytosol as it was formulated in the signal hypothesis and led to discovery of the fundamental principal concepts of protein sorting [10,11,12,13]. Surprisingly, signal peptides do not have substantial amino acid homology but possess common structural elements. General organization of signal peptide includes n, h, and c regions (Figure 1). The N-terminal (n-region) is about 1–5 amino acid residues, usually has a positive charge due to the presence of one or several basic amino acids, the hydrophobic core (h-region) is a stretch of 7–15 hydrophobic amino acid residues, and the carboxy-terminal region (c-region, 3–7 amino acid residues) is more polar and contains cleavage site for signal peptidase [14,15]. Cleavage sites are described by the (−3, −1) rule, where small neutral amino acid residues are located in position −3 and only Ala, Gly, Ser, Cys, Thr, Gln are in position −1 of the signal sequence (−1 position is the last amino acid residue in the signal sequence) [15,16]. Interestingly, eukaryotic and prokaryotic signal peptides have a similar organization. The specific structure of the signal sequence and proper coordination of the secretory protein synthesis is important for their biogenesis. Imbalance in amount of secretory proteins due to their elevated synthesis (overproduction, for instance) leads to their precursor aggregation [17,18]. In bacteria, mutations in the h-region of signal peptides may dramatically inhibit the protein ability to be secreted (incorporation of polar amino acid residues in the h-region, for instance), changes in the n-region are more tolerable but mutations may influence secretion efficiency, while alterations in the c-region may inhibit processing [19,20,21]. In mammals, the mutations in the signal sequence may interfere with interaction with SRP, where the mutations in the hydrophobic core play a critical role [22]. Importantly, mutations decreasing hydrophobicity of the h-region of the mammalian signal sequences lead to specific inhibition of the mutant protein synthesis and their mRNA template degradation (see below for details) [8,23,24].

### 2.2. Signal Recognition Particle (SRP) Binds Signal Peptides and Targets Ribosomes to the ER Membrane

**Figure 2:**
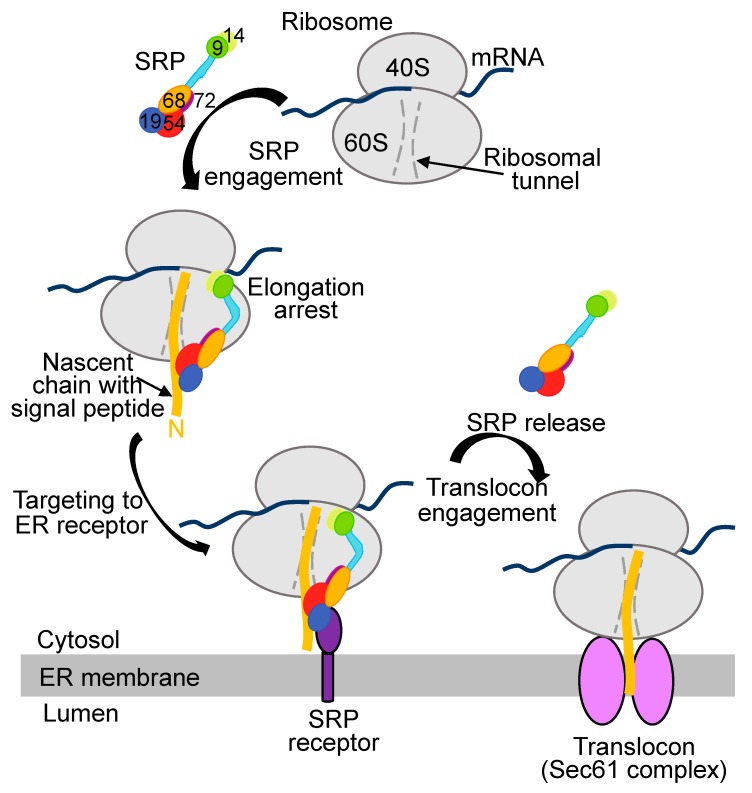
Signal Recognition Particle (SRP) pathway for targeting secretory proteins to the endoplasmic reticulum (ER) membrane. Signal recognition particle is a multiprotein complex composed of a non-coding RNA (7SL RNA) and six protein subunits, SRP9 (green), SRP14 (yellow), SRP68 (orange), 72 (dark purple), SRP19 (blue) and SRP54 (red). All protein subunits are assembled on the about 300 nucleotide long 7SL RNA (light blue). When a secretory protein is being synthesized on ribosome, SRP recognizes its N-terminal signal peptide, binds it, temporary stops translation and targets the whole ribosome-nascent chain complex (RNC) to the ER membrane. SRP receptor located in the ER membrane binds SRP-RNC complex. Interactions with the receptor triggers engagement of Sec61 translocon with consequent release of the targeting factor (SRP). Nascent chain now is co-translationally translocated in the ER lumen where processing and modifications of new protein occur.

In mammals, signal peptides are recognized co-translationally by a targeting factor SRP [2]. However, bacterial counterpart of SRP recognizes inner membrane proteins, while SecA, a protein that is not found in eukaryotes, recognizes signal sequences of secretory proteins [25,26]. It seems that there are two targeting factors in bacteria, SecA and bacterial SRP, that are able to interact with their substrates co-translationally [25,26,27,28,29]. Bacterial SRP consists of Ffh (fifty-four-homolog) protein and 4.5S RNA [30]. Eukaryotic SRP is a multi-subunit complex that consists of six proteins, SRP9, SRP14, SRP19, SRP54, SRP68, SRP72, and one non-coding RNA, 7S RNA (7SL RNA) [3]. This complex is divided in two large domains, S (signal peptide binding) and Alu (named for the presence of *Alu* sequence in this part of 7S RNA). SRP binds signal peptide immediately after they emerged from the nascent polypeptide tunnel at the ribosome [2,31,32,33]. SRP54 subunit that is located in the S domain of the SRP complex directly binds signal sequences [31,32]. Mammalian SRP has a very high affinity to the nascent chains with signal sequences on the ribosome (0.05–0.38 nM) [34]. When SRP binds the nascent chains containing signal peptides, it changes its conformation from extended to L-shape positioning the S domain near the nascent chain exit and the Alu domain near the binding site for elongation factors [35] (Figure 2). This arrangement prevents elongation of the nascent chain leading to temporal elongation arrest. This is an important event. First, it prevents appearance of the potentially dangerous secretory proteins in the cytosol, and, second, protects the proteins from possible misfolding in the cytosol because they need ER chaperones for their proper folding. Formation of the ribosome-nascent chain-SRP complex leads to its targeting to the SRP receptor (SR) in the ER membrane, GTP hydrolysis, transferring the ribosome-nascent chain complex to the translocon and the release of the SRP (Figure 2). The ribosomes resume the protein synthesis and the polypeptide nascent chains are co-translationally transported through the translocon into ER lumen, signal peptides are cleaved off by membrane bound signal peptidase, some nascent peptides are glycosylated, and transported further through Golgi outside of the cells or some proteins are remained in the ER or associated with membrane. The fine details of these processes may be found in several publications [3,36,37,38].

## 3. Quality Control of mRNAs and Proteins during Translation

**Figure 3:**
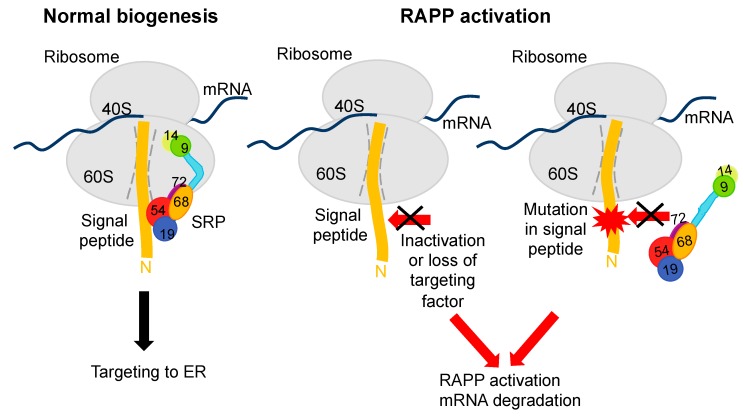
Regulation of Aberrant Protein Production (RAPP) pathway. During normal biogenesis, SRP functions co-translationally as a targeting factor for delivery of secretory proteins into ER. If interactions between signal peptide of the secretory proteins and SRP are disrupted due to inactivation of SRP, loss of targeting factor or mutations in a signal peptide, then RAPP is activated and mRNA of the secretory proteins is degraded [8,23,24]. SRP subunits are labeled by numbers and colors as in Figure 1.

mRNA and protein quality controls are very important processes directed to remove or prevent synthesis of potentially toxic products in the cells. Several systems exist to degrade defective proteins. Among them are cytosolic ubiquitin/proteasome system, endoplasmic reticulum associated degradation (ERAD), and the unfolded protein response (UPR) [39,40,41,42,43,44]. These pathways sense and destroy misfolded or aberrant proteins that are already synthesized and need to be removed. However, the most intriguing protective quality control mechanisms are those that work on the level of protein synthesis during mRNA translation when proteins are being synthesized and, thus, these pathways are ribosome-associated or translation driven quality controls [1,45,46]. Several mechanisms were identified that control mRNA quality – NMD (nonsense-mediated decay), NGD (no-go-decay), NSD (non-stop decay) to remove faulty mRNAs with premature stop-codons, or those mRNAs that stalled during translation or with missing natural stop codon, respectively [45,47,48,49,50]. The aberrant truncated polypeptides produced from these defective mRNAs are marked by ubiquitin and degraded by proteasome in the process now known as RQC (ribosome quality control) [46,51,52,53,54]. These systems are relatively well studied and there are several excellent reviews published recently that describe protein and RNA surveillance in the cells, protein degradation and these mechanisms in detail [55,56,57,58,59,60,61,62,63,64]. In addition to these pathways, the secretory proteins are controlled by a unique mechanism that we named Regulation of Aberrant Protein Production (RAPP) [8]. This pathway monitors interactions of polypeptide nascent chains during their synthesis on the ribosomes and degrades their mRNAs if these interactions are abolished due to mutation in the nascent chains or defects in interacting partner (Figure 3). While the pathway is likely to control surveillance of different types of proteins, currently it was demonstrated only for secretory proteins [8,23,24,65]. Originally, it was discovered on the example of preprolactin with deletions of leucines from its hydrophobic core [8]. It was demonstrated that deletion of one, two, three and four leucine leads to gradual decrease in interaction with SRP, increase of the nascent chain crosslinking to AGO2 protein, and corresponding decrease in mRNA levels of the mutated proteins. The degradation of defective proteins mRNAs was specific to the mutant forms only and no effect on the wild-type mRNAs was found when they were co-expressed. The specific degradation of the secretory protein mRNAs was observed when the SRP54 subunit was depleted in the cultured human cells. Thus, the mechanism is able to select and degrade the mRNAs of proteins that were not able to interact with SRP regardless of the cause of the interaction loss (due to mutations in the nascent chains or due to defects in SRP). Many new substrates for the RAPP pathway were discovered and it was demonstrated that its pathological activation is a molecular mechanism of many human diseases [23,24,65]. However, the fine molecular mechanism of RAPP is still unknown. The role of AGO2 in the process is not understood. AGO2 was found in close proximity to the mutated preprolactin nascent chains and even to the wild-type protein nascent chain when SRP was defective (SRP54 knockdown). AGO2 depletion suppressed the mutant prolactin mRNA degradation, while AGO2 overexpression stimulated it [8]. However, AGO2 depletion and overexpression did not affect the mRNA level of the mutated A9D granulin, the other RAPP substrate [23]. These data suggest that during RAPP, AGO2 acts as a sensor for some defective proteins and some other factor may conduct that function for other aberrant proteins. AGO2 is known for its function in post-translational gene silencing (RNA interference, RNAi), and it is a subunit of the RISC (RNA induced silencing complex) [66,67,68,69]. It has a ribonuclease H or slicer activity [70,71,72]. Surprisingly, AGO2 ribonuclease enzymatic activity is not required for the RAPP pathway. It was also found that Dicer and Drosha, proteins involved in miRNA biogenesis, and miRNAs are not involved in the RAPP process [8]. These data suggest a novel Ago2 function leaving question about nature of the RNase in RAPP opened. Despite the lack of the details of the mechanism, the data obtained on examples of many mutated secretory proteins demonstrate that RAPP is a general pathway protecting cells from accumulation of the potentially toxic mislocalized proteins in the cytosol by degrading their mRNAs.

## 4. Defective SRP, Mutations in Secretory Proteins and Human Diseases

Protein targeting and secretion are fundamental processes. Many secretory proteins conduct essential functions for cell viability. Thus, many human diseases associated with secretory defects were found. It was shown recently that mutations in the SRP54 subunit cause neutropenia and Shwachman-Diamond-like syndrome [73,74]. Eight different mutations in total were identified in two studies: three mutations (T115A, T117del, G226E) in one work [73], and seven (G113R, T117del, C118Y, C136Y, A223D, G226E, G274D) in the other [74] (Table 1). The mutations are located in the SRP54 GTPase domain (G domain). The molecular mechanism of the mutation-associated diseases in these cases is most likely connected with reduced GTP hydrolysis and the SRP receptor binding. Indeed, it was found that all three mutations (T115A, T117del, G226E) in recombinant SRP54 reduce the GTPase activity [73]. The SRP54 GTPase activity is essential for protein targeting. The complex formation between SRP and SRP receptor promotes each other GTPase activity, providing efficient targeting of secretory proteins and dissociation of the complex [75]. The mutations in the SRP54 GTPase domain make this process inefficient resulting in the disease. Two autosomal-dominant mutations in the other SRP subunit, SRP72, were found associated with familial aplasia and myelodysplasia, one was a missense mutation R207H, the other led to frameshift and resulted in truncated version of the protein [76] (Table 1). SRP is also associated with other human diseases where anti-SRP autoantibody is produced in patients (polymyositis, severe myositis, interstitial lung disease, necrotizing myopathy, and other diseases) [77,78,79,80,81,82,83,84]. SRP-co-translational translocation is up-regulated in lung cancer [85]. It was demonstrated that 7SL RNA transferred by exosomes to breast cancer cells activates tumor growth and metastasis [86]. There are also many other human diseases that are associated with protein transport defects downstream of the protein synthesis (see for details [87,88,89,90,91]). However, they are not in the scope of this review that focuses on events regulated on the ribosome during translation.

**Figure 4:**
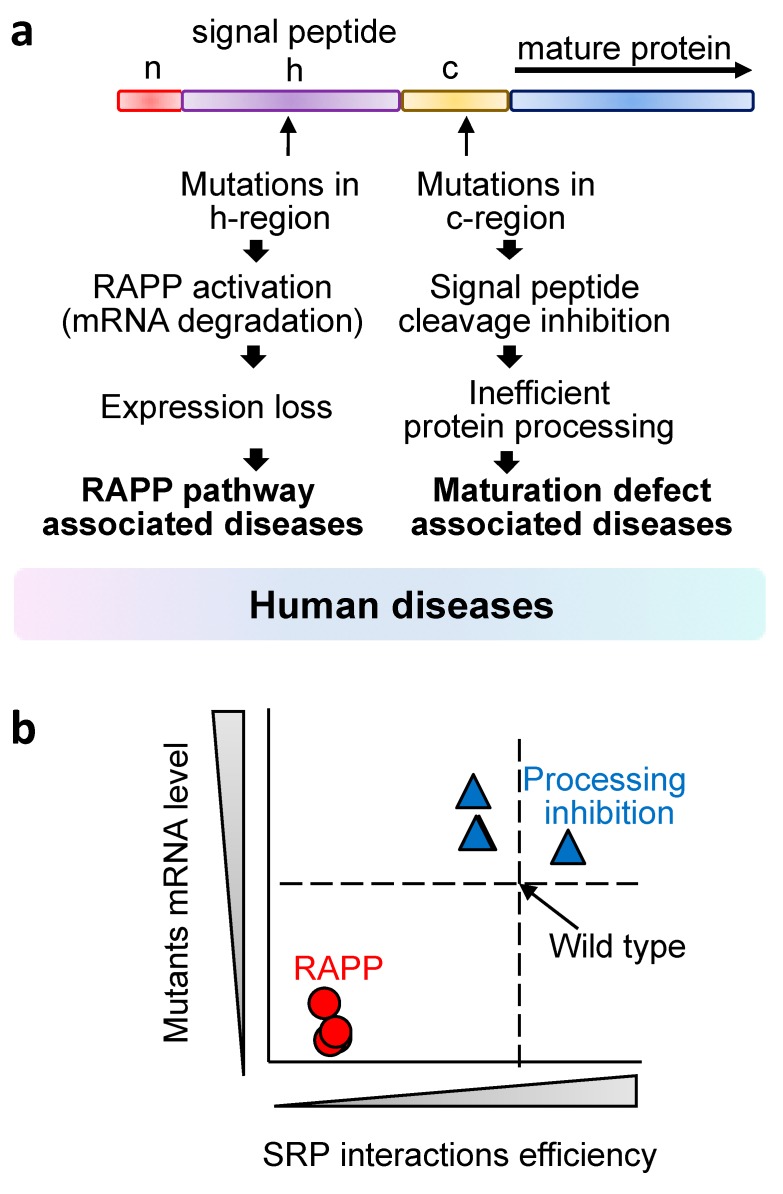
Mutations in signal sequences and human diseases. (**a**) Locations of mutations in the signal sequences and possible molecular mechanisms of human diseases. (**b**) Graph representation of the effects of the mutations in signal sequences on the mRNA level (mRNA stability) and on SRP – nascent chain interactions. When a mutation is located in h-region of a signal peptide and affects its hydrophobic properties, it leads to the loss of interactions with SRP and decrease of mRNA level by the triggering the RAPP pathway activation (red circles). The outcome of this pathway is a degradation of mRNAs of defective secretory proteins. If a mutation is located in the c-region of a signal peptide and does not inhibit interaction with SRP and does not lead to mRNA degradation (blue triangles) it may affect maturation of the protein due to the failure of signal sequence cleavage by signal peptidase. Please note that a benign mutation (natural polymorphism) not associated with a disease will show a similar plot.

Diseases associated with mutations in the signal peptides of secretory proteins represent an important group of disorders for understanding their underlying molecular mechanisms [23,24,65,92]. This group constitutes of very diverse diseases because secretory proteins conduct wide variety of functions, and disruption of one of them may have very different consequences from disruption of the other protein. Some examples of disease-associated mutations in the signal sequences are presented in Table 2. Although the diseases are very different, their molecular mechanisms are determined by the type and position of mutations in the signal sequences. It was known for a long time that mutations changing hydrophobicity of the hydrophobic core (h-region) inhibit protein transport in vitro. These observations led to assumption that the molecular mechanism of the diseases associated with the mutations in that region is the inhibition of protein translocation through ER membrane. However, recently we demonstrated on the example of many secretory proteins that these types of disease-associated mutations activate the RAPP pathway [23,24]. The mutated signal sequences were not able to be recognized by SRP, and mRNAs of these secretory proteins were specifically degraded and no proteins were expressed. Thus, the lack of expression due pathological activation of the RAPP pathway is the most likely scenario for some familial types of frontotemporal lobar degeneration, some forms of aspartylglucosaminuria, pycnodysostosis, and others [23,24,65]. Interestingly, the level of mRNA degradation in the RAPP pathway depends on the severity of the mutation in the h-region and the nature of the signal peptide as well. In general, alteration of hydrophobic amino acid residues for charged amino acid residues (positively charged Arg, negatively charged Asp) and helix breaker Pro residues leads to a very strong RAPP response - dramatic mRNA reduction and loss of expression of mutated granulins (GRN W7R, GRN A9D), aspartylglucosaminidase (AGA L15R), and cathepsin K (CTSK L7P, CTSK L9P) (Table 2). Substitution of hydrophobic Val for hydrophobic Leu in a natural variant of granulin (GRN V5L) does not inhibit signal sequence interaction with SRP and does not activate the RAPP pathway and, thus, represents a harmless gene polymorphism and is not associated with the disease [23,65]. Mutations in the signal peptide c-region do not inhibit interaction with SRP and do not induce the RAPP pathway suggesting a different molecular mechanism of the diseases in that cases (see LIPA and COL10A1 proteins for example) [24]. We proposed that diseases-associated mutations in signal peptides may be caused by varied mechanisms, or even combination of the mechanisms [24] (Figure 4a). If mutations disrupt interaction with SRP, they activate the RAPP pathway that leads to the mutated mRNA degradation and protein expression loss. These mutations are located in the h-region and decrease hydrophobicity of the signal peptide. Thus, pathological RAPP activation is the molecular mechanism of these types of diseases. However, if mutations are located in the c-region, they may prevent or inhibit cleavage of the signal peptide, and, thus, the molecular mechanism of the diseases may be associated with inefficient maturation. These observations allow predicting molecular mechanisms of newly found disease-associated mutations by analyzing their position in the signal peptide and hydrophobicity profiles. Graph shown in Figure 4b demonstrates visually the distinction of these two molecular mechanisms by plotting the SRP interactions efficiency and mutant mRNA levels. Both of these mechanisms are engaged co-translationally when the nascent chain is still being synthesized. The RAPP pathway is activated in the very beginning of protein synthesis when the mutated signal peptide just appears from the exit of the polypeptide tunnel on the ribosome, while the processing defects occur at the late stages of translation when the mutated cleavage site is exposed to the signal peptidase on the luminal side of the ER membrane and the enzyme is not able to cleave off the signal sequence because of the mutations.

## 5. Conclusions and Perspectives

The goal of this review is to provide analysis and a brief discussion of major events in the biogenesis of secretory proteins that are controlled on the ribosome during translation and regulate expression, targeting, and even stability of their mRNA templates. The crucial element for all these processes is a balance of interactions of the nascent chain with its partners at the ribosome exit site. Effective SRP interaction with signal peptides is a key element for the secretory protein expression, transport, and their mRNA stability. Thus, SRP has a dual function, first, in protein targeting, and second, in mRNA protection from degradation. Weakening of SRP and secretory protein interaction due to mutations in signal peptide or defects in SRP initiates the RAPP protective mechanism to prevent synthesis and release of the defective protein in the cytosol. The RAPP pathway specifically degrades the mRNAs of the secretory proteins when this interaction is inefficient. The degree of the mRNA degradation depends on the severity of the mutation, thus finely adjusting the expression level with ability to translocate the defective proteins. When mutations appear in the signal peptides of secretory proteins, they may lead to their expression loss and cause diseases. It is demonstrated that the pathological activation of the RAPP pathway due to mutations in secretory proteins results in many human diseases. Because secretory proteins have very diverse functions, their expression loss may be associated with different types of human diseases as it was shown on many examples. Although the general concept of the RAPP pathway and its association with human diseases came into sight, its mechanistic details are still unknown and need to be addressed in the future studies.

## Figures and Tables

**Table 1:** Disease-associated mutations in SRP subunits.

SRP Subunit	Mutation	Disease	References
SRP54	G113R, T115A, T117del, C118Y, C136Y, A223D, G226E, G274D	Neutropenia and Shwachman-Diamond-like syndrome	[73,74]
SRP72	R207H, truncated T355K due to two nucleotides deletion and frameshift	Aplasia (aplastic anemia), myelodysplasia	[76]

**Table 2:** Human diseases associated with mutations in signal peptides of secretory proteins ^1^.

Gene (Protein)	Signal Sequence Plus 2 Amino Acid Residues (Cleavage Site is Underlined) ^2^	Mutation	mRNA Expression ^3^	Disease or Note	References
*GRN* (granulin)	MWTLVSWVALTAGLVAG TR MWTLVSWV**D**LTAGLVAG TR MWTLVS**R**VALTAGLVAG TR MWTL**L**SWVALTAGLVAG TR	Wild-type A9D W7R V5L	+++++ ++ + +++++	Frontotemporal lobar degeneration (FTLD); V5L is a benign polymorphism	[23]
*AGA* (aspartylglucosaminidase)	MARKSNLPVLLVPFLLCQALVRC SS MARKSNLPVLLVPF**R**LCQALVRC SS	Wild-type L15R	+++++ +	Aspartylglucosaminuria	[24,93]
*CTSK* (cathepsin K)	MWGLKVLLLPVVSFA LY MWGLKV**P**LLPVVSFA LY MWGLKVLL**P**PVVSFA LY	Wild-type L7P L9P	+++++ + +	Pycnodysostosis	[24,94,95]
*UGT1A1* (UDP-glucuronosyltransferase)	MAVESQGGRPLVLGLLLCVLGPVVS HA MAVESQGGRPLVLG**R**LLCVLGPVVS HA	Wild-type L15R	+++++ ++	Crigler-Najjar disease	[24,96]
*SERPINA7* (serpin peptidase inhibitor A7)	MSPFLYLVLLVLGLHATIHC AS MSPFLYLVLLVLGLHATI**Y**C AS	Wild-type H19Y	+++++ ++	Thyroxine-binding globulin deficiency	[24,97]
*NDP* (Norrie disease protein)	MRKHVLAASFSMLSLLVIMGDTD SK MRKHVLAASFSM**R**SLLVIMGDTD SK MLSLLVIMGDTD SK	Wild-type L13R Δ11	+++++ +++ +++	Norrie disease	[24,98]
*PTH* (parathyroid hormone)	MIPAKDMAKVMIVMLAICFLTKSDG KS MIPAKDMAKVMIVMLAI**R**FLTKSDG KS MIPAKDMAKVMIVMLAICFLTK**P**DG KS	Wild-type C18R S23P	+++++ +++ +++	Hypoparathyroidism	[24,99,100]
*TGFB1* (transforming growth factor beta 1)	MPPSGLRLLPLLLPLLWLLVLTPGRPAAG LS MPPSGLRLL**L**LLLPLLWLLVLTPGRPAAG LS MPPSGLRLLPLLLPLLWLLVLTPG**P**PAAG LS	Wild-type P10L R25P	+++++ ++++ +++	Renal function decline, osteoporosis, proliferative diabetic retinopathy	[24,101,102,103,104]
*CTLA4* (cytotoxic T-lymphocyte associated protein 4)	MACLGFQRHKAQLNLATRTWPCTLLFFLLFIPVFC KA MACLGFQRHKAQLNLA**A**RTWPCTLLFFLLFIPVFC KA	Wild-type T17A	+++++ ++++	Autoimmune disease	[24,105]
*LHB* (luteinizing hormone beta polypeptide)	MEMLQGLLLLLLLSMGGAWA SR MEMLQGLLLLLLLSMGG**T**WA SR	Wild-type A18T	+++++ ++++	Hypogonadotropic hypogonadism	[24,106]
*SERPINE1* (serpin peptidase inhibitor E1)	MQMSPALTCLVLGLALVFGEGSA VH MQMSPALTCLVLGL**T**LVFGEGSA VH	Wild-type A15T	+++++ ++++	Fibrinolytic bleeding disorder	[24,107]
*PRSS1* (serine protease 1)	MNPLLILTFVAAALA AP MNPLLILTFVAAALA **V**P	Wild-type A16V	+++++ +++++	Chronic pancreatitis	[24,107,108]
*COL10A1* (collagen type X alpha 1)	MLPQIPFLLLVSLNLVHG VF MLPQIPFLLLVSLNLVH**R**VF MLPQIPFLLLVSLNLVH**E**VF	Wild-type G18R G18E	+++++ +++++	Schmid metaphyseal chondrodysplasia	[24,109,110]
*LIPA* (lipase A)	MKMRFLGLVVCLVLWPLHSEGSG GKL MKMRFLGLVVCLVLWPLHSEGS**R** GKL	Wild-type G23R	+++++ +++++	Wolman disease	[24,111]
*PRL* (prolactin, bovine)	MDSKGSSQKGSRLLLLLVVSNLLLCQGVVS TP MDSKGSSQKGSRLLLLLVVSNLLCQGVVS TP MDSKGSSQKGSRLLLLLVVSNLCQGVVS TP MDSKGSSQKGSRLLLLVVSNLCQGVVS TP MDSKGSSQKGSRLLLVVSNLCQGVVS TP	Wild-type Δ1L Δ2L Δ3L Δ4L	+++++ ++++ +++ ++ +	Artificial mutations	[8,22]

^1^ Selected signal sequence mutations and relevant diseases are shown as examples. ^2^ Missense mutations are marked by red bold font and underlined. **^3^** Approximate mRNA expression levels where wild-type mRNA is taken as the highest mRNA level (+++++), while (+) is a lowest mRNA expression level.

## Abbreviations

SRP	Signal Recognition Particle
ER	Endoplasmic reticulum
ERAD	Endoplasmic reticulum associated degradation
UPR	Unfolded protein response
NMD	Nonsense-mediated decay
NGD	No-go-decay
NSD	Non-stop decay
RAPP	Regulation of Aberrant Protein Production
